# Valorization of Spent Brewer’s Yeast by Pulsed Electric Field Treatment Combined with Enzymatic Hydrolysis

**DOI:** 10.3390/jof12040250

**Published:** 2026-03-31

**Authors:** Valentina Ganeva, Boyana Angelova

**Affiliations:** Department of Biophysics and Radiobiology, Faculty of Biology, Sofia University “St. Kliment Ohridski”, 1164 Sofia, Bulgaria; angelova_bd@uni-sofia.bg

**Keywords:** pulsed electric field, irreversible electropermeabilization, spent brewer’s yeast, Alcalase, enzymatic hydrolysis, peptides, antioxidant activity, phenolic content

## Abstract

Spent brewer’s yeast, a major by-product of the brewing industry, is a valuable source of bioactive compounds. The main technological limitation for their recovery is the rigid yeast cell wall, while the high nucleic acid content may restrict the direct use of yeast-derived extracts for human nutrition. In this study, pulsed electric field (PEF) treatment, applied alone or in combination with enzymatic hydrolysis, was investigated for the production of yeast-derived extracts with different compositions. PEF treatment performed in continuous-flow mode resulted in more than 98% of cells with irreversibly permeabilized membranes and enabled the rapid and selective release of low-molecular-weight intracellular compounds during subsequent incubation of the cells in water. Within 4 h, approximately 61% of total antioxidant activity, 65% of glutathione, and around 80% of free α-amino nitrogen and B-group vitamins were recovered at different rates, while the aqueous extracts were characterized by low purine nucleotide content. Electropermeabilized cells exhibited high sensitivity to enzymatic hydrolysis. After 6 h of incubation with 0.2% (*v*/*v*) Alcalase, the obtained hydrolysates contained 254 ± 17 mg/g DCW of protein, mostly in the form of peptides, 148.2 ± 17.3 mg/g DCW of free α-amino nitrogen, and a total phenolic content of 16.7 ± 1.9 mg GAE/g DCW. The maximal antioxidant activity (62.7 ± 9.3 mg TE/g DCW) was reached after 4 h of incubation, corresponding to a 2.7-fold increase compared with cell lysates. Overall, PEF treatment, applied alone or in combination with enzymatic hydrolysis, provides an efficient and mild approach for the production of yeast-derived extracts with tailored compositions and potential applications in the food, pharmaceutical, and cosmetic industries.

## 1. Introduction

Brewer’s yeast is produced in large quantities as one of the main by-products of the brewing industry and is generally regarded as safe (GRAS). It is a rich source of proteins, which typically represent 45–55% of the cell biomass, with a high proportion of essential amino acids [1,2,3]. In addition, it contains substantial amounts of minerals, B-group vitamins, antioxidants, phenolic compounds, β-glucans, and other bioactive constituents, which makes it a promising ingredient for use in nutraceutical, cosmetic, and pharmaceutical formulations [4,5]. Despite this valuable composition, its current exploitation is still rather limited, and the predominant use of spent brewer’s yeast is as an inexpensive animal feed and fertilizer, usually after heat inactivation [2,6].

There are two main factors that restrict the use of whole cells in the human diet, both as a health supplement and as a protein source. The first is the very rigid, indigestible cell wall, which decreases the bioavailability of intracellular compounds and may provoke allergies and gastrointestinal disorders [7]. Another important limitation is the high nucleic acid content of spent brewer’s yeast (6–15% of dry weight), which consists predominantly of RNA [8,9]. These nucleic acids are rich in purine bases, which, upon digestion, are metabolized to uric acid. Because humans lack the enzyme uricase, purine degradation results in uric acid accumulation, and excessive uric acid levels are associated with various metabolic disorders [4,6]. Therefore, the use of spent brewer’s yeast as a source of bioactive compounds for human consumption requires cell-wall disruption and a substantial reduction in nucleic acids.

Spent brewer’s yeast is the main raw material for the production of yeast extracts used in a wide range of applications, including fermentation medium components, animal feed additives, flavoring agents, and sources of bioactive compounds for the food, pharmaceutical, and cosmetic industries [6,9,10,11,12]. Yeast extract is generally defined as the soluble fraction obtained after treatments that affect the integrity of the cell envelope (plasma membrane and cell wall), thereby allowing the release of various cellular constituents. Such treatments include mechanical disruption, autolysis, hydrolysis with exogenous enzymes, or combinations of these methods [9,10,11,12,13]. The composition and, consequently, the functional properties of yeast extracts strongly depend on the nature of the raw material as well as on the processing methods applied during their production.

In recent years, growing attention has focused on extraction strategies that produce peptide-enriched extracts, as such peptides exhibit a wide range of bioactive properties [11,14]. Depending on their molecular weight, amino acid composition, and physicochemical characteristics, these peptides can enhance the antioxidant capacity of yeast extracts and confer additional biological activities, such as antihypertensive, immunomodulatory, anti-ulcer, and antiproliferative effects [10,11,15,16,17]. Consequently, peptide-rich yeast extracts represent promising ingredients for nutraceutical and pharmaceutical products, with increasing interest in cosmetic applications due to their antioxidant and skin-protective properties [9,11,18].

One of the most efficient methods for obtaining yeast extracts enriched in biologically active peptides is enzymatic hydrolysis, using either a single exogenous endopeptidase or a mixture of enzymes applied simultaneously or sequentially to maximize solids recovery and tailor extract characteristics. In practice, the enzymes most commonly used for yeast hydrolysis are commercial preparations such as Protamex™, Flavourzyme™, Alcalase™, and Brauzyn^®^ [11,12,15,19,20].

Since a large proportion of yeast proteins are located inside the cell, effective hydrolysis requires exogenous enzymes to overcome the cell envelope, including the plasma membrane and the cell wall. Yeasts possess a relatively thick cell wall with a complex, layered structure [21]. In addition to its high resistance to mechanical disruption, mainly determined by the inner glucan layer, the yeast cell wall exhibits very limited permeability to macromolecules due to the outer layer, which consists of highly glycosylated, tightly packed mannoproteins [22]. This structure significantly hinders the access of extracellular enzymes to intracellular compounds and thereby reduces the efficiency of enzymatic hydrolysis. This barrier becomes even more pronounced after serial repitching cycles (up to six), commonly applied in brewing, resulting in decreased cell wall porosity and consequently limiting protease access to intracellular proteins [20].

To enhance the efficiency of enzymatic hydrolysis, additional processing steps are therefore often required. These include autolysis, applied either as a pretreatment or simultaneously with enzymatic hydrolysis [9,23]; mechanical disruption prior to enzymatic hydrolysis [24]; and pretreatment with cell wall-degrading enzymes [11,20]. While these strategies generally improve hydrolysis efficiency, each additional treatment step increases process complexity. Autolysis requires prolonged incubation at elevated temperatures and may lead to partial loss of biological activity of thermolabile compounds, reduced process efficiency, and an increased risk of microbial contamination [25]. Mechanical disruption, although effective and widely applied at the industrial scale, causes extensive cell fragmentation, complicates downstream separation and fractionation, and is associated with high energy demand [26,27]. The use of multiple enzymes, including those with cell wall-degrading activities, is often required to overcome these limitations, but this further increases process complexity and cost.

Accordingly, an alternative pretreatment that disrupts the integrity of the plasma membrane and increases cell wall porosity could provide a promising route toward more efficient enzymatic hydrolysis of spent brewer’s yeast and improved overall process performance.

Pulsed electric field (PEF) treatment has been widely reported as a non-thermal, energy-efficient, and scalable technology for enhancing the recovery of bioactive compounds from microorganisms and plant tissues [28,29]. Its effect is primarily associated with a loss of plasma membrane barrier properties, commonly referred to as electroporation or electropermeabilization, resulting from the generation of an additional transmembrane potential [30,31]. Depending on the electrical parameters (field strength, pulse number, and pulse duration), cell characteristics (size and physiological state), and the composition of the pulsing medium, the loss of membrane barrier function may become irreversible. Irreversible electropermeabilization is associated with extensive leakage of ions and water-soluble intracellular molecules of various molecular sizes and ultimately leads to cell death [32,33].

As a rule, in cells possessing a cell wall (e.g., plant cells and microorganisms), irreversible electropermeabilization does not result in cell fragmentation, thereby enabling milder and more selective extraction of intracellular constituents [29,34,35]. However, extraction efficiency, particularly for macromolecules such as proteins, is largely governed by the cell wall, which may significantly limit their release, depending on its composition and structure [34,36,37]. In this context, several strategies have been applied. One approach involves combining PEF with externally supplied cell-wall-degrading enzymes to increase cell wall permeability and facilitate the release of proteins from microbial systems with very strong and impermeable cell walls, including microalgae [38] and yeast [35]. The applied electric field alone can induce structural modifications in the cell wall of different cell types [35,39,40,41]. Under suitable electrical conditions, yeast cell wall porosity increases significantly, thereby enabling the release of large intracellular and periplasmic enzymes [42]. In addition, these PEF-induced structural changes markedly enhance the efficiency of cell-wall-degrading enzymes [35,42].

Another strategy involves applying PEF to increase cell susceptibility to exogenous proteases. For example, electrical treatment leading to irreversible permeabilization has been shown to enhance the enzymatic hydrolysis of fresh biomass from the microalga *Scenedesmus almeriensis* by enabling the entry of Alcalase and Flavourzyme into the cells [43]. More recently, a similar approach was shown to significantly improve the enzymatic hydrolysis of baker’s yeast, enabling rapid recovery of intracellular compounds and the generation of extracts with high antioxidant activity [44]. PEF treatment may represent a promising strategy to improve enzyme access to intracellular proteins, particularly in yeast cells with thick and relatively impermeable cell walls, without the need for mechanical disruption, prolonged incubation at elevated temperatures, or the addition of cell wall-degrading enzymes. This approach may contribute to faster hydrolysis under milder conditions and facilitate downstream processing.

In the present study, we evaluate the recovery of bioactive compounds from spent brewer’s yeast following irreversible electropermeabilization and demonstrate, for the first time, that PEF pretreatment significantly enhances cell susceptibility to the exogenous endoprotease Alcalase. This combined approach enables the rapid and efficient production of extracts enriched in valuable intracellular constituents.

## 2. Materials and Methods

### 2.1. Yeast Biomass

The spent brewer’s yeast used in this study was kindly provided by Kamenica AD (Haskovo, Bulgaria). All experiments were performed using cell biomass of *Saccharomyces pastorianus* obtained after three to four repitching cycles. The yeast biomass was first collected by centrifugation (2600× *g*, 10 min), and the supernatant consisting of residual fermentation medium was discarded. The cell pellet was washed once with distilled water, resuspended in distilled water, and incubated for 1 h at room temperature. After a second centrifugation step (2600× *g*, 10 min), the biomass was resuspended in distilled water to a final concentration of 63 ± 3.7 g dry cell weight per liter (g DCW/L). The conductivity of the suspension was then adjusted to 0.30 ± 0.02 mS/cm using a 0.25 M sodium phosphate buffer at pH 7.

### 2.2. Pulsed Electric Field Treatment

Pulsed electric field (PEF) treatment in a continuous-flow chamber was performed using a generator of monopolar rectangular pulses (2300 V–10 A), Hydropuls mini (GBS-Elektronik, Radeberg, Germany), as previously described [45]. Pulse duration and frequency were controlled by an arbitrary waveform generator (RIGOL DG1012, Suzhou, China). The treatment chamber (volume, 0.825 mL) has two parallel stainless-steel electrodes spaced 0.4 cm apart. The PEF treatment was conducted at flow rates of 35 mL/min and 140 mL/min, controlled by a peristaltic pump (Ismatec, Glattbrugg, Switzerland). During passage through the chamber, the cells were exposed to trains of pulses with durations ranging from 0.025 to 0.5 ms and electric field strengths between 2 and 5.5 kV/cm. The total treatment time (t) was calculated as the product of the number of pulses (n) and the pulse duration (τ), according to the equation: t = n × τ. The specific treatment energy per liter of cell suspension was calculated as previously described [45].

All electrical parameters were monitored online with an oscilloscope (Instek GDS 2064, New Taipei City, Taiwan). At flow rates of 35 and 140 mL/min, the residence times of the cells in the treatment chamber were approximately 1.41 s and 0.35 s, respectively. The outlet temperature was recorded using a K-type thermocouple connected to a digital thermometer, with the sensor positioned at the outlet of the chamber.

### 2.3. Determination of Dead Cells and Irreversible Electropermeabilization

The fractions of dead cells prior to electrical treatment and cells with irreversibly permeabilized plasma membranes after PEF treatment were determined using propidium iodide (PI) staining. To assess cell viability before PEF treatment, 5 µL of a 0.5 mM PI solution in distilled water was added to 50 µL of cell suspension. The samples were incubated for 5 min at room temperature, washed once with distilled water, and examined using an epifluorescence microscope (L3201 LED, Microscopesmall, Shenzhen, China). The number of fluorescent cells was counted and expressed as a percentage of the total cell population.

A similar protocol was applied to determine irreversible membrane permeabilization after PEF treatment. In this case, PI staining was performed 1 h after electrical treatment. The degree of permeabilization was expressed as the percentage of fluorescent cells relative to the total cell count.

### 2.4. Lyticase Test

Electroinduced changes in cell wall porosity were assessed using a lyticase assay. Pulsed and untreated (control) cell suspensions were diluted in phosphate buffer (PPB, pH 7.5) to a final buffer concentration of 167 mM and a cell concentration of 21 mg dry cell weight per milliliter (mg DCW/mL). Lyticase (Sigma-Aldrich Chemie GmbH, Taufkirchen, Germany) was added to a final concentration of 80 U/mL, and the suspensions were incubated at 35 °C for 90 min. Subsequently, 8 µL of each sample was added to 1000 µL of distilled water, and the optical density (OD) at 660 nm was measured. A decrease in OD directly reflected the extent of cell lysis. OD values were expressed as percentages relative to the OD measured before enzyme addition, which was taken as 100%.

### 2.5. Post-PEF Processing of Cell Suspension

After pulsed electric field (PEF) treatment, both PEF-treated and untreated (control) cell suspensions were processed in parallel under identical post-treatment conditions, depending on the subsequent analysis.

#### 2.5.1. Release of Water-Soluble Compounds

To analyze the release of intracellular water-soluble compounds, both PEF-treated and untreated (control) cell suspensions were incubated at room temperature without further dilution. Samples were collected at 15, 30, and 60 min during the first hour of incubation and subsequently at hourly intervals up to 4 h (2, 3, and 4 h), as well as at 6, 8, and 20 h, and centrifuged for 2 min at 10,750× *g* (Eppendorf centrifuge) or for 10 min at 2600× *g* (ROTINA 380, Hettich, Tuttlingen, Germany). The supernatants were analyzed for different compounds immediately or stored at −20 °C until further analysis.

#### 2.5.2. Enzymatic Hydrolysis

Enzymatic hydrolysis was performed using Alcalase^®^ 2.4 L FG, kindly provided by Novozymes (Bagsværd, Denmark). For this procedure, 25 mL of PEF-treated and control cell suspensions were transferred into 250 mL Erlenmeyer flasks and mixed with an equal volume of 100 mM K_2_HPO_4_, resulting in an initial pH of 8.5. The flasks were equilibrated for 15 min at 40 °C, after which Alcalase was added to a final concentration of 0.2% (*v*/*v*). The suspensions were incubated at 40 °C with shaking at 100 rpm in a Biosan ES-20/40 orbital shaker (Biosan, Riga, Latvia). Samples were collected after 1, 2, 4, and 6 h, centrifuged, and immediately frozen at −20 °C until further analysis.

### 2.6. Preparation of Cell Lysate

Cell lysis was performed by mechanical disruption using glass beads (diameter 0.42–0.6 mm, Sigma-Aldrich Chemie GmbH, Steinheim, Germany). Briefly, 2 mL of cell suspension, diluted twofold in distilled water or 100 mM K_2_HPO_4_ solution, was mixed with glass beads in a plastic capped tube at a suspension-to-beads ratio of 2:1 (*v*/*v*). The samples were vortexed for nine cycles of 1 min each, with 15 s pause intervals between cycles, during which the tubes were incubated on ice. The resulting lysates were centrifuged at 10,750× *g* for 5 min, and the supernatants were collected and stored at 4 °C until analysis.

### 2.7. Analytical Methods

#### 2.7.1. Total Protein and Free α-Amino Nitrogen (FAN) Determination

Total protein was determined according to Lowry et al. [46], with bovine serum albumin as a standard. Free α-amino nitrogen (FAN) was determined using a ninhydrin-based colorimetric assay [47]. Briefly, the ninhydrin reagent was prepared by dissolving ninhydrin in a solvent mixture containing DMSO and 0.25 M sodium acetate buffer (pH 5.5) at a ratio of 40:60 (*v*/*v*), to a final concentration of 0.2% (*w*/*v*). In microcentrifuge tubes, 200 μL of the amino-acid-containing sample solution was mixed with 200 μL of the ninhydrin reagent. The tubes were incubated in a dry block heater at 90 °C for 15 min. After cooling, 1 mL of an ethanol–water solution (1:1, *v*/*v*; prepared from 97% ethanol) was added, followed by vortex mixing. Absorbance was measured at 570 nm. FAN content was expressed as mg glycine equivalents per g dry cell weight (mg GE/g DCW), based on a calibration curve constructed with glycine (10–100 mg/L) as a standard.

#### 2.7.2. Trolox Equivalent Antioxidant Capacity (TEAC) and Reduced Glutathione (GSH)

Total antioxidant activity was determined by the TEAC (Trolox equivalent antioxidant capacity) assay using 2,2′-azino-bis(3-ethylbenzothiazoline-6-sulfonic acid) diammonium salt (ABTS), as described by Pellegrini et al. [48] with slight modifications. The ABTS^•+^ stock solution (7 mM aqueous solution of ABTS with 2.45 mM potassium persulfate) was diluted with 50 mM potassium phosphate buffer pH 7 to an absorbance of 0.70 ± 0.02 at 734 nm. The samples (5 µL) of two- to four-fold diluted extracts were mixed with 995 µL of working solution. The decrease in absorbance at 734 nm was measured after 15 min. The Trolox standard curve was determined in the range of 0.1–15 µM. The data are expressed as mg Trolox equivalent per gram of cell dry weight (mg TE/g DCW).

Reduced glutathione (GSH) was determined by a colorimetric method using 5,5′-dithiobis-(2-nitrobenzoic acid) (DTNB), according to Rahman et al. [49], with modifications. Briefly, 20 µL of a 0.2% (*w*/*v*) DTNB solution prepared in 0.1 M potassium phosphate buffer (PPB; pH 7.5) containing 5 mM EDTA was added to 960 µL of the same buffer. Subsequently, 20 µL of sample (supernatant of electrically treated and control cell suspensions or the soluble fraction of the cell lysate obtained after mechanical disintegration) was added, and the reaction mixtures were incubated for 2–10 min at room temperature. Absorbance was measured at 412 nm in triplicate. GSH concentration was calculated using a calibration curve constructed with reduced L-glutathione as a standard.

#### 2.7.3. Total Phenolic Content (TPC)

Total phenolic content (TPC) was determined according to Singleton et al. [50], with modifications. To 50 µL of appropriately diluted supernatants from electrically treated and control cell suspensions or cell lysate, 750 µL of Folin–Ciocalteu reagent (previously diluted tenfold with distilled water) and 700 µL of 7.5% (*w*/*v*) Na_2_CO_3_ were added. The reaction mixtures were incubated at 30 °C for 30 min, and absorbance was measured at 765 nm. A standard curve constructed with known concentrations of gallic acid (GA) was used to calculate the total phenolic content, expressed as mg gallic acid equivalents per g dry cell weight (mg GAE/g DCW).

#### 2.7.4. Determination of Purines and B-Vitamins

Purine content was determined spectrophotometrically as described by Makkar and Becker [51]. The amounts of released pyridoxine (vitamin B_6_) and niacin (vitamin B_3_) were determined using standard microbiological methods according to the manufacturer’s instructions for Pyridoxine Y Medium and Niacin Assay Medium (Difco Laboratories, Becton Dickinson, Sparks, MD, USA). *Saccharomyces cerevisiae* 1083 and *Lactobacillus plantarum* 1009 (National Bank for Industrial Microorganisms and Cell Cultures, Sofia, Bulgaria) were used as auxotrophic test organisms for pyridoxine and niacin determination, respectively. For each analysis, a standard curve was prepared, and after incubation, cell growth was measured spectrophotometrically at 660 nm. Vitamin concentrations in the samples were calculated by comparison with the standard curves.

#### 2.7.5. The Tricine-Sodium Dodecyl Sulfate Polyacrylamide Gel Electrophoresis

The tricine-sodium dodecyl sulfate polyacrylamide gel electrophoresis (Tricine-SDS-PAGE) of the protein samples was performed on 14.7% polyacrylamide slab gels, as described by Schägger [52]. Proteins were detected by silver staining, following the method of Nesterenko et al. [53], and the protein molecular weight markers used were PageRuler™ Prestained Protein Ladder (10–170 kDa, Thermo Fisher Scientific, Waltham, MA, USA).

### 2.8. Determination of Biomass and Soluble Solid Content

Dry cell weight was determined by microwave drying as described by Rice et al. [54]. Aliquots of 1 mL of cell suspension were filtered through membrane filters (pore size 0.45 µm), and the filters containing the retained biomass were dried in a microwave oven to constant weight. Dry cell weight was calculated as the difference between the filter weight before and after filtration and drying. The soluble solids released from PEF-treated cells incubated in water were determined indirectly by comparing the dry cell weight of control and electropermeabilized cells after 4 h of incubation, as described above.

Changes in fresh cell weight were determined 2 and 4 h after electrical treatment. Aliquots of 1 mL of PEF-treated and control cell suspensions were transferred to pre-weighed Eppendorf tubes and centrifuged at 10,750× *g* for 2 min. The supernatant was carefully removed, and the tubes containing the cell pellets were weighed again. Fresh cell weight was calculated as the difference between the weight of the tube containing the cell pellet and the weight of the empty tube. The fresh weight of control cells was set to 100%, and the fresh weight of PEF-treated cells was expressed relative to this value.

The solid content of yeast hydrolysate was determined after 6 h of incubation with the enzyme. A total of 20 mL of cell suspension from control and PEF-treated cells were centrifuged (2600× *g*, 15 min), and the supernatants were transferred to pre-weighed glass vessels. The samples were dried at 100 °C to constant weight. The released soluble solids were calculated as the difference between the weight of the glass vessels before and after drying. The values were corrected for the contribution of K_2_HPO_4_ present in the suspension.

### 2.9. Statistical Analysis

All experiments were performed in 3–5 independent biological replicates, each measured in at least triplicate (technical replicates). For each biological replicate, the mean of the technical measurements was calculated. Data are presented as mean ± standard deviation (SD) of the biological replicates. Statistical analysis was performed using Microsoft Excel (Microsoft Office 2021). Differences between groups were evaluated using a Student’s *t*-test, and *p* < 0.05 was considered statistically significant.

## 3. Results and Discussion

### 3.1. Release of Water-Soluble Substances

For high-efficiency extraction of intracellular compounds, irreversible permeabilization of the plasma membrane is required. Therefore, various combinations of electrical parameters were initially tested, with total treatment times ranging from 2 to 5 ms and electric field strengths between 2 and 5.5 kV/cm. Electrical treatment was applied at a constant flow rate of 35 mL/min. For subsequent experiments, two parameter combinations resulting in irreversible plasma membrane permeabilization in more than 98% of the treated cells were selected: (i) 10 pulses of 500 µs duration at 7.1 Hz (total treatment time, 5 ms), field strength of 3.2 kV/cm, outlet temperatures of 36–38 °C; (ii) 90 pulses of 25 µs duration, at 63.6 Hz (total treatment time, 2.25 ms), field strength of 5.0 kV/cm, outlet temperatures of 30–32 °C. Subsequently, PEF-treated and control cell suspensions were incubated at room temperature for up to 20 h. At selected incubation times, cells were separated by centrifugation, and the supernatants were collected for analysis.

Yeast antioxidant potential is determined by different soluble and insoluble cell components. Approximately 85–90% of the free radical scavenging activity in the soluble fraction obtained after mechanical cell disruption is attributed to compounds with a molecular mass below 10 kDa [55]. Among these, the predominant low-molecular-weight intracellular antioxidant is glutathione, which plays a key role in redox homeostasis, together with soluble phenolic compounds that contribute to the overall antioxidant capacity.

**Figure 1:**
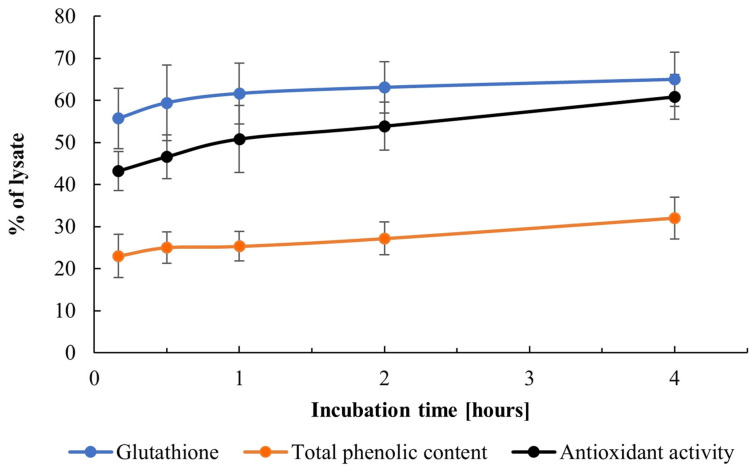
Release of glutathione, phenolic compounds, and antioxidant activity from PEF-treated cells. Electrical treatment conditions were as follows: electric field strength 3.2 kV/cm, 10 pulses of 0.5 ms duration, and pulse frequency of 7.1 Hz. Data represent mean ± SD (*n* = 3).

In the present study, glutathione was released predominantly during the first minutes after PEF treatment, exceeding 61% of the total glutathione content determined in the lysate (6.37 ± 0.29 mg/g DCW) within 1 h (Figure 1). After 4 h of incubation, this value increased slightly to approximately 65% and remained stable for up to 20 h. For comparison, the glutathione content in extracts from control cells reached only 0.59 ± 0.17 mg/g DCW, indicating limited spontaneous release in the absence of PEF treatment.

Enhancement of glutathione release from permeabilized brewer’s yeast incubated in alkaline buffer was recently reported [56]. The present data demonstrate that comparable extraction efficiency can be achieved by incubating PEF-treated cells in water, which is consistent with previous results obtained for baker’s yeast [45]. The observed differences may reflect variations in cell biomass composition as well as in the electrical parameters applied for cell permeabilization.

A rapid release of phenolic compounds was observed during the first hours of incubation, reaching approximately 30% of the total content (9.06 ± 0.53 mg GAE/g DCW) within 4 h (Figure 1). The release continued more slowly thereafter, attaining 46.6% after 20 h of incubation. The relatively low extraction efficiency can be attributed to the fact that a portion of the phenolic compounds is bound to cell wall constituents or intracellular macromolecules, and their release usually requires treatment with organic solvents and/or buffer solutions [57]. Therefore, under the conditions applied in this study, only the soluble phenolic fraction is expected to be released.

The antioxidant capacity of the extracts reflected the combined contribution of these low-molecular-weight compounds. During the first 4 h, approximately 61% of the total activity present in the cell lysate (21 ± 2.2 mg TE/g DCW) was released (Figure 1). Thereafter, the release rate decreased markedly, reaching 13.3 ± 0.7 mg TE/g DCW after 20 h, corresponding to approximately 63.3% of the total. These release kinetics are consistent with previous findings in baker’s yeast, where the antioxidant capacity of aqueous extracts originated mainly from compounds smaller than 10 kDa, including glutathione and phenolics [45].

Treatment with 25-µs pulses (total treatment time of 2.25 ms) at 5.0 kV/cm yielded extracts with essentially the same content of glutathione, phenolic compounds and antioxidant activity as treatment with 500-µs pulses (total treatment time of 5 ms) at 3.2 kV/cm, despite the differences in the outlet temperatures observed.

**Figure 2:**
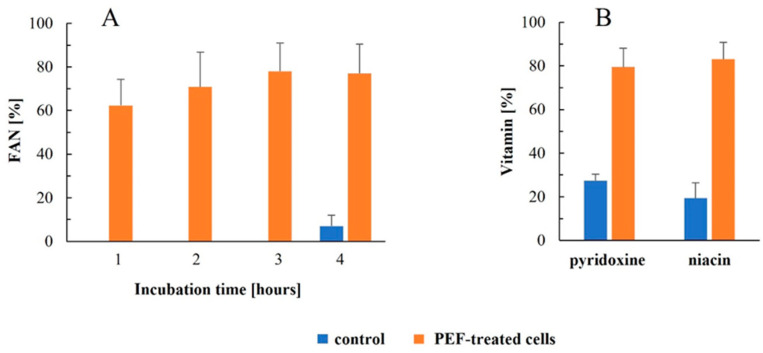
Free α-amino nitrogen (FAN) release from control and PEF-treated cells after 1–4 h of incubation at room temperature (**A**) and release of B-group vitamins (pyridoxine and niacin) after 3 h of incubation (**B**). Electrical treatment conditions were as follows: electric field strength 5.0 kV/cm, 90 pulses of 25 µs duration, and pulse frequency of 63.6 Hz. Data are presented as mean ± SD (*n* = 3). Values are expressed as a percentage of the total content obtained after mechanical disruption of control cells.

Analysis of free α-amino nitrogen (FAN), a parameter reflecting the content of free amino acids and peptides, revealed a rapid release during the first hour of incubation. After 4 h, the FAN content in the extracts from PEF-treated cells reached 77 ± 13.5% of the level obtained after mechanical disruption (37 ± 5 mg/g DCW) (Figure 2A). A similar extraction efficiency (72 ± 3%) was achieved when the cells were treated with 10 pulses of 0.5 ms at 3.2 kV/cm. These results are consistent with earlier reports on baker’s yeast incubated in water after PEF treatment [45] and on PEF-treated spent brewer’s yeast incubated in alkaline buffer [56].

Spent brewer’s yeast is characterized by a high content of B vitamins and is therefore considered a valuable natural source of these micronutrients, with potential applications in dietary supplements and cosmetic formulations [11,18]. To evaluate the suitability of PEF treatment for recovering these compounds, we monitored the release of two representative water-soluble vitamins, niacin and pyridoxine, 1–4 h after pulse application. Both vitamins rapidly diffused into the medium, reaching a plateau at approximately 80–83% of their intracellular content by 3 h (Figure 2B). The 100% reference value corresponds to the vitamin content in lysates obtained after mechanical disruption of the cells (1.53 ± 0.35 mg pyridoxine and 40.6 ± 7 mg niacin per 100 g DW). The pyridoxine levels obtained in the present study for the cell lysate are lower than those reported in the literature [19], which may reflect natural variation in yeast biomass composition or the use of alternative analytical methods.

**Figure 3:**
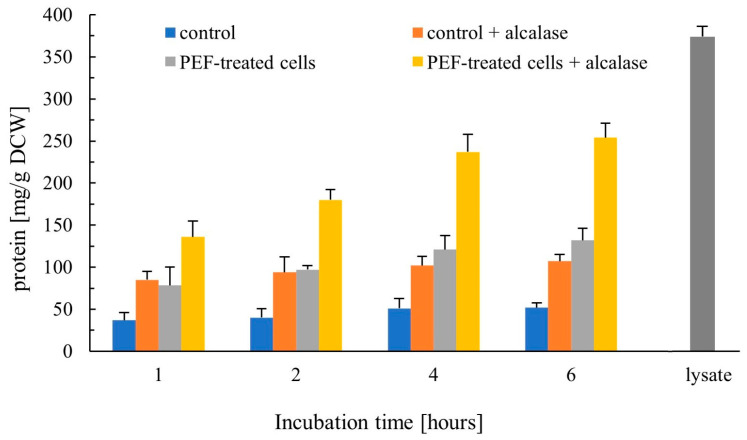
Effect of Alcalase on protein release from control and PEF-treated cells. Control and PEF-treated cell suspensions were incubated in 50 mM K_2_HPO_4_ with or without 0.2% (*v*/*v*) Alcalase for up to 6 h at 40 °C, and released protein was quantified at the indicated time points. The protein content in the cell lysate obtained after mechanical disruption is shown as a reference. Data are presented as mean ± SD (*n* = 4).

The protein content in the extracts obtained after 4 h of incubation of permeabilized cells in water reached 38 ± 9 mg/g DCW for both sets of electrical parameters. This represents only a minor fraction of the total cellular protein, which was 374 ± 12 mg/g DCW upon complete cell disruption (as shown in Figure 3). A previous study has shown that irreversible electropermeabilization of yeast suspensions at a similar cell concentration in distilled water results in rapid (≈10 min) release of intracellular ions and stabilization of the suspension pH at around 5.5 [45]. Given that the isoelectric point of most cytosolic proteins in yeast lies between pH 4.5 and 6 [58], the combined effect of intracellular acidification and decreased ionic strength may promote partial aggregation of soluble cytosolic proteins during subsequent incubation in water, leading to their retention by the cell wall.

The amount of purines released under the same conditions was 2.76 ± 0.19 mg/g DCW, which is approximately ten times lower than the total purine content reported for brewer’s yeast in the literature [59]. This observation confirms previous findings indicating that, when permeabilized yeast cells are incubated in water at room temperature, nucleic acids remain largely retained inside the cells, while endogenous RNases exhibit very low or no activity, resulting in minimal release of purine nucleotides [45].

The soluble solids in the aqueous extracts were determined indirectly by comparing the dry weight of the cell biomass before and after PEF treatment, followed by 4 h of incubation at room temperature, as described in Section 2. The yield reached 172.5 ± 17.4 mg/g DCW, confirming that the process primarily enables the release of low-molecular-weight, water-soluble intracellular compounds. In addition to the components analyzed in this study, these compounds are expected to include inorganic ions, small carbohydrates, organic acids, and other low-molecular-weight metabolites naturally present in the cytosol.

It has been shown that irreversible electroporation of yeast cells in water is associated with a reduction in cell size [60]. Yeast cells typically contain 65–75% water, depending on growth conditions and physiological state. Here, we compared the fresh weights of control and electrically treated samples two hours after treatment and incubation at room temperature. PEF treatment led to a 37.5 ± 3% (*n* = 4) decrease in yeast biomass fresh weight. Prolonged incubation did not result in any further reduction. Considering the amount of soluble solids released, this decrease in fresh weight mainly reflects water loss. This dewatering effect arises from the rapid efflux of ions and small osmolytes following irreversible plasma membrane permeabilization in a hypotonic medium and is most probably further enhanced by mechanical stress and compaction during centrifugation.

### 3.2. Enzymatic Hydrolysis of PEF-Treated Cells

During incubation of permeabilized cells in water, most proteins and nucleic acids, as well as a fraction of phenolic compounds, remain confined within the cells, and their release therefore requires additional treatments depending on the target molecules and the intended application of the resulting yeast extract. Enzymatic hydrolysis with exogenous proteases is widely used to obtain peptide-rich yeast extracts; however, its efficiency strongly depends on the ability of the enzymes to cross the yeast cell wall.

Previous studies have demonstrated that PEF treatment under conditions leading to irreversible plasma membrane permeabilization also increases cell wall porosity, even in yeast strains characterized by a highly compact wall structure [35]. Sensitivity to lytic enzymes is widely accepted as an indicator of structural modifications in the outer mannoprotein layer, which controls permeability to macromolecules under different physiological and processing conditions [61,62]. Accordingly, we compared the response of control and PEF-treated cells to lyticase.

The electrical conditions applied were as described in the previous section (10 pulses of 0.5 ms duration, electric field strength of 3.2 kV/cm, and a flow rate of 35 mL/min). After 1 h of incubation with 80 U/mL lyticase, the OD_660_ of control cells decreased to 85.0 ± 4.7% (*n* = 3), whereas that of PEF-treated cells declined much more markedly to 37.4 ± 5.6% (*n* = 3). This pronounced difference confirms that PEF treatment substantially enhances the permeability of spent brewer’s yeast cell walls to macromolecules, in agreement with previous findings for other yeast species [35].

In subsequent experiments, we examined the effect of PEF pretreatment on the enzymatic hydrolysis of yeast cells using Alcalase, a serine endopeptidase derived from *Bacillus licheniformis*. This enzyme was selected because of its high effectiveness in protein hydrolysis and the high antioxidant capacity of the extracts obtained [20,63]. Moreover, we expected that its relatively low-molecular-weight (27.3 kDa) would facilitate diffusion through the yeast cell wall and improve access to intracellular protein substrates, as previously reported for baker’s yeast [44]. Cells were exposed to 10 pulses of 0.5 ms duration at a frequency of 28.3 Hz and a flow rate of 140 mL/min. The pulse frequency was adjusted to achieve a total treatment time of 5 ms under these conditions. Irreversible permeabilization in more than 98% of the treated cells was obtained at electric field strengths of 3.1–3.2 kV/cm. The outlet temperature remained between 36 and 38 °C, and the specific treatment energy was 62.9 ± 4.3 kJ/L (*n* = 4).

The permeabilized cell suspensions were diluted twofold with 100 mM K_2_HPO_4_, resulting in an initial suspension pH of 8.5, and Alcalase was added to a final concentration of 0.2% (*v*/*v*). The samples were incubated at 40 °C for different time intervals as described in Section 2. This temperature provides, on the one hand, sufficiently high enzymatic activity [64], and on the other, milder incubation conditions that prevent thermal inactivation or degradation of various bioactive components.

Typically, extracts obtained after enzymatic hydrolysis are analyzed after heat inactivation of the enzyme. However, heating can cause thermal unfolding and precipitation of a fraction of the proteins present in the extracts, leading to an underestimation of their actual protein content and altering their composition. Therefore, to obtain a representative profile of the extracts and to allow direct comparison with those obtained without Alcalase, the samples were analyzed immediately after thawing, without heat inactivation. Protein measurements were corrected using control samples containing the same concentration of Alcalase.

#### 3.2.1. Protein Release

Permeabilized cells released a considerable amount of protein even in the absence of enzyme (Figure 3). Incubation of the PEF-treated cells with Alcalase further increased protein release, yielding more than twice the amount obtained from the enzyme-treated control cells. After 6 h, the released protein reached 254 ± 17 mg/g DCW, corresponding to approximately 70% of the level achieved by mechanical disruption (374 ± 12 mg/g DCW). This marked increase can be attributed to the improved access of Alcalase to intracellular protein substrates as a result of plasma membrane permeabilization and enhanced cell wall porosity, as observed previously [44]. Considering that the protein content of spent brewer’s yeast typically accounts for 45–55% of the dry cell weight, the combined PEF–enzymatic treatment enables the release of approximately half of the available protein within a relatively short incubation period.

**Figure 4:**
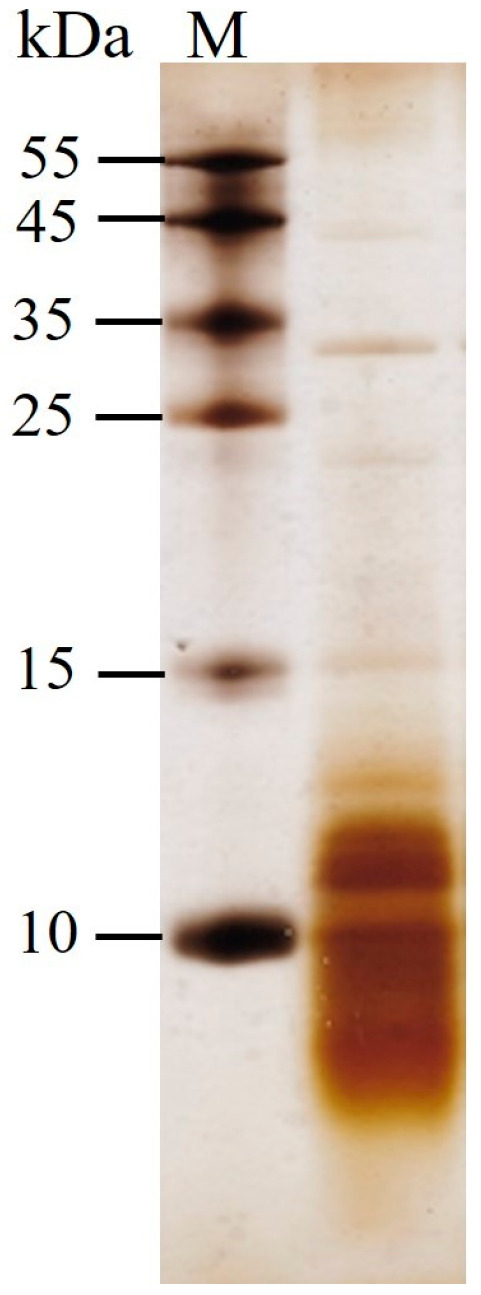
Tricine-SDS-PAGE analysis of proteins released from PEF-treated cells after 6 h incubation with Alcalase. Lane M: molecular weight marker.

Overall, protein release from PEF-treated spent brewer’s yeast was considerably more efficient than that reported for pressed baker’s yeast under similar conditions [44]. This difference may reflect structural variations in the cell wall, either strain-specific characteristics or changes resulting from repeated reuse and fermentation conditions. The presence of 11.5 ± 1.3% non-viable cells after 3–4 repitching cycles could also contribute to the protein detected in the control samples, as well as to the greater susceptibility of the control cells to Alcalase compared with baker’s yeast. As shown by Tricine–SDS–PAGE analysis, the extracts obtained from PEF-treated cells incubated with Alcalase contained mainly peptides of less than 15 kDa (Figure 4). Due to the limited porosity of the yeast cell wall, protein release from electropermeabilized cells is generally a slow process, even when cells are incubated in a buffer at an optimal pH of 8–8.5 and in the presence of thiol compounds that increase cell wall permeability by disrupting disulfide bonds in the outer mannoprotein layer [42]. Achieving a protein yield of ~70–80% of that obtained after mechanical disruption typically requires prolonged incubation of electropermeabilized cells for 20–48 h at 30 °C [45,56]. The present results demonstrate that combining PEF treatment with Alcalase enables a comparable extraction efficiency to be reached within 4–6 h. This improvement can be attributed to the facilitated access of Alcalase to intracellular protein substrates and to the rapid diffusion of the hydrolysis products through the cell wall. The predominance of peptides below 15 kDa may also reflect a degree of size-selective release. In this sense, the cell wall may function as a natural filtration system, contributing to the fractionation and partial purification of peptides during the hydrolysis process.

#### 3.2.2. Free α-Amino Nitrogen (FAN) Content

**Figure 5:**
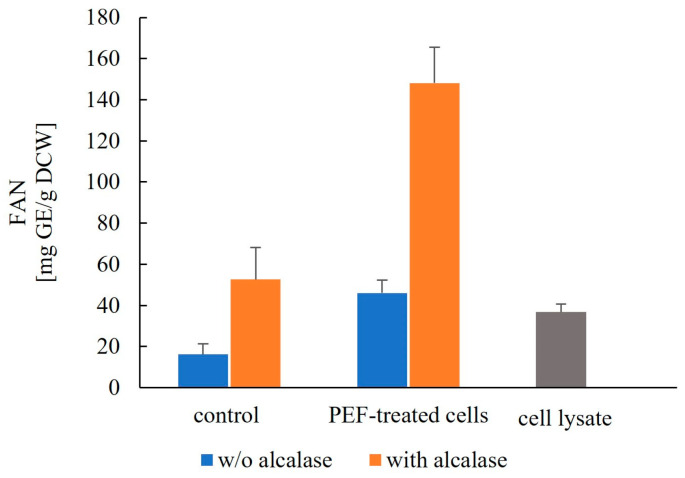
Effect of Alcalase on FAN release from control and PEF-treated cells after 6 h of incubation at 40 °C. Data are presented as mean ± SD (*n* = 3).

The free α-amino nitrogen (FAN) content of the extracts is shown in Figure 5. Even in the absence of Alcalase, extracts from PEF-treated cells exhibited higher FAN values than the lysate (*p* < 0.01), indicating that partial protein hydrolysis occurred during incubation at 40 °C over this relatively short period. This effect can be attributed to the activity of endogenous proteases released as a consequence of vacuolar destabilization due to PEF treatment [56,65].

Electropermeabilization markedly enhances enzymatic hydrolysis, with FAN levels reaching 148.2 ± 17.3 mg/g DCW, i.e., 2.8-fold higher than in control cells incubated with Alcalase and fourfold higher than in cell lysates. Overall, these substantially elevated FAN values demonstrate that combining PEF pretreatment with enzymatic hydrolysis enables significantly faster and more extensive proteolysis of spent brewer’s yeast than that achieved by enzymatic hydrolysis or PEF treatment when applied individually.

#### 3.2.3. Release of Phenolic Compounds

**Figure 6:**
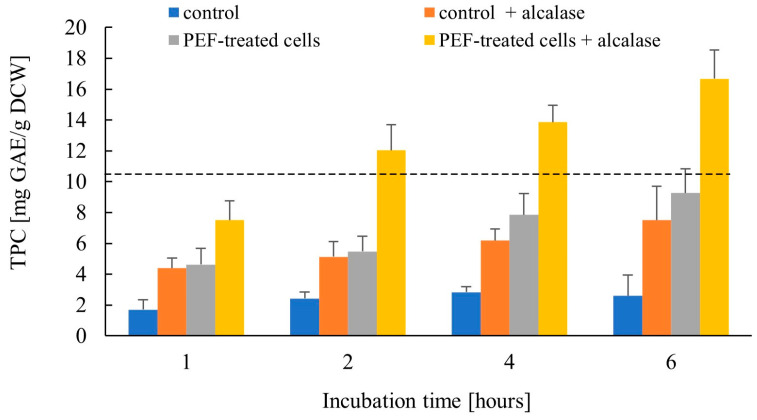
Effect of Alcalase on the release of phenolic compounds from control and PEF-treated cells. Control and PEF-treated cell suspensions were incubated in 50 mM K_2_HPO_4_ with or without 0.2% (*v*/*v*) Alcalase for up to 6 h at 40 °C and total phenolic content (TPC) was determined at the indicated time points. The TPC in the cell lysate obtained after mechanical disruption is shown as a dashed line. Data represent mean ± SD (*n* = 4).

During successive fermentation cycles, yeast biomass accumulates considerable amounts of phenolic compounds from the brewing medium [66]. These bioactive constituents substantially contribute to the antioxidant capacity of spent brewer’s yeast extracts. Although a fraction of yeast phenolics, such as catechin and gallic acid, exists in soluble form, a portion remains bound within the cells, associated with the cell wall and intracellular macromolecules [57], which limits their release into aqueous media after plasma membrane permeabilization. Here, we evaluated the release of phenolic compounds from control and PEF-treated yeast cells after incubation at 40 °C for various time intervals, in the presence and absence of Alcalase (Figure 6).

The total phenolic content (TPC) detected in the extracts reflects the combined contribution of several processes acting simultaneously. In control cells incubated without enzyme, the relatively low yet detectable phenolic levels are most likely due to desorption of non-covalently bound compounds from the cell wall in the alkaline medium [57]. The addition of Alcalase to control cells resulted in a time-dependent increase in phenolic content, most likely due to partial hydrolysis of cell wall mannoproteins and the release of associated phenolics. In electropermeabilized cells incubated without Alcalase, the detected phenolics originate from intracellular leakage and enhanced desorption of wall-bound compounds, presumably favored by structural changes induced in the cell wall by PEF treatment. When PEF-treated cells were incubated with Alcalase, a pronounced enhancement in phenolic release was observed, reaching 16.7 ± 1.9 mg GAE/g DCW after 6 h, corresponding to approximately 160% of the TPC measured in the cell lysate (10.4 ± 1.3). This value was more than twice that obtained for control cells treated with the enzyme (7.5 ± 2.5 mg GAE/g DCW), indicating that PEF-assisted enzymatic hydrolysis promotes the liberation of phenolics associated with both cell wall and intracellular structures, in addition to those released by PEF-induced membrane permeabilization and cell wall destabilization alone.

Phenolic compounds are usually recovered from yeast by organic solvent extraction, alkaline treatment, autolysis, or mechanical disruption, and the efficiency of these methods also depends on the cell pretreatment, such as lyophilization or debittering, which may lead to a reduction in phenolic content [25,57,66,67]. PEF treatment, often in combination with organic solvent extraction, has been extensively shown to enhance phenolic recovery from various plant matrices, including spent brewer’s grain [28,29,68]. Recently, we demonstrated that PEF treatment alone enables partial recovery of phenolic compounds from baker’s yeast [35], and that the combination of PEF and Alcalase further improves phenolic extraction [44]. The present results demonstrate that PEF treatment followed by enzymatic hydrolysis markedly increases the recovery of phenolics from spent brewer’s yeast, yielding values higher than those commonly reported for conventional extraction methods [25,57,67]. Nevertheless, it should be noted that phenolic content may vary depending on the yeast strain and the composition of the brewing medium.

#### 3.2.4. Release of Antioxidant Activity and Soluble Solids

**Figure 7:**
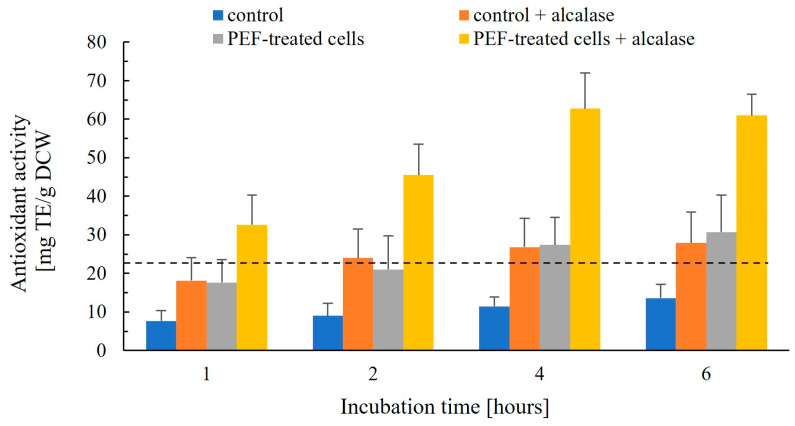
Effect of Alcalase on the antioxidant activity released from control and PEF-treated cells. Control and PEF-treated cell suspensions were incubated in 50 mM K_2_HPO_4_ with or without 0.2% (*v*/*v*) Alcalase at 40 °C for up to 6 h, and the released antioxidant activity was determined at the indicated time points. The antioxidant activity in the cell lysate obtained after mechanical disruption is shown as a dashed line. Data are presented as mean ± SD (*n* = 3).

The release of antioxidant activity from PEF-treated and control cells followed a pattern comparable to that observed for phenolic compounds (Figure 7). A moderate increase was detected in extracts from control cells incubated with Alcalase and from PEF-treated cells without enzyme, where the antioxidant activity reached or slightly exceeded the value measured for the cell lysate prepared in 50 mM K_2_HPO_4_ (22.9 ± 2.9 mg TE/g DCW).

In contrast, incubation of PEF-treated cells with Alcalase resulted in a pronounced enhancement, with maximal antioxidant activity observed after 4 h of incubation, exceeding 270% of the lysate value, followed by a slight decline upon prolonged incubation. This behavior differed both in magnitude and kinetics from phenolic release. After 4 h of enzymatic treatment, total phenolic content reached approximately 133% of the lysate value and continued to increase with extended incubation. Thus, at the same time point, the relative increase in antioxidant activity was approximately two-fold greater than that of total phenolic content. The observed differences in the efficiency of antioxidant activity and phenolic release from PEF-treated cells incubated with Alcalase suggest that, in addition to phenolic compounds, enzymatic hydrolysis of intracellular and cell wall-associated proteins substantially contributes to the detected antioxidant activity through the formation of bioactive peptides and free amino acids with high radical-scavenging capacity. Yeast proteins are enriched in hydrophobic and aromatic amino acid residues, which are commonly associated with antioxidant and ACE-inhibitory activities of derived peptides [10]. Moreover, although Alcalase exhibits broad substrate specificity, it preferentially cleaves peptide bonds in hydrophobic regions [63], which may account for the pronounced increase in antioxidant activity. The synergistic effect between cell electropermeabilization and enzymatic hydrolysis therefore represents an efficient strategy for obtaining antioxidant-rich yeast extracts.

The soluble solid content of the extract obtained from PEF-treated cells incubated with Alcalase for 6 h reached 51.7 ± 5.4% (*n* = 3) of the dry cell weight. This solid recovery is comparable to that reported for spent brewer’s yeast subjected to combined enzymatic hydrolysis with Protamex (0.6%) and Flavourzyme (2%) for 12 h at 50 °C [12]. The ability to achieve similar extraction yields under milder conditions and shorter processing times suggests potential advantages of the combined PEF–enzymatic treatment.

#### 3.2.5. Effect of Heat Treatment on Protein, Total Phenolic Content, and Antioxidant Activity in Extracts and Cell Lysates

In these experiments, we compared the composition of extracts obtained from PEF-treated cells after 6 h of incubation with Alcalase with that of cell lysates obtained by mechanical disruption, before and after heat inactivation. Samples were subjected to heat treatment immediately after thawing (for frozen extracts) or directly after preparation (for cell lysates). Aliquots were transferred to microcentrifuge tubes and incubated at 90 °C for 15 min in a temperature-controlled heating block. After heating, the samples were centrifuged at 10,750× *g* for 2 min, and the supernatants were collected for subsequent analysis of protein content, total phenolic content, and antioxidant activity.

**Figure 8:**
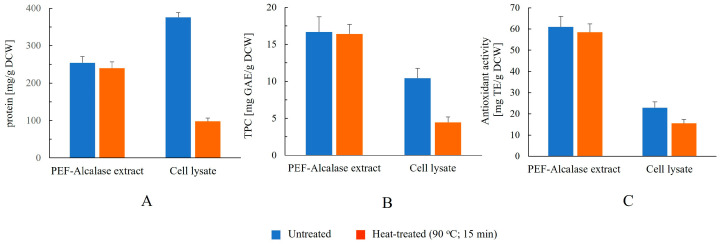
Effect of heat treatment on protein (**A**), total phenolic content (**B**), and antioxidant activity (**C**) in extracts obtained from PEF-treated cells after 6 h of incubation with Alcalase and cell lysates. Data represent mean ± SD (*n* = 3).

As shown in Figure 8, the extracts produced by the combined treatment contain predominantly thermostable components, as no significant differences were detected in protein content, total phenolic content, or antioxidant activity after heating. The absence of a decrease in protein content can be explained by the predominance of short peptides (<10–15 kDa), as demonstrated by SDS–PAGE analysis (Figure 4). Unlike intact globular proteins, such peptides are less susceptible to heat-induced denaturation. Phenolic compounds are also generally stable under the applied heating conditions, which further contributes to the preservation of overall antioxidant activity.

In contrast, heating of lysates obtained after mechanical disruption resulted in a pronounced reduction in protein content, consistent with thermal denaturation and precipitation of proteins. A substantial decrease in total phenolic content was also observed, indicating that a portion of the phenolics released during mechanical disruption remains associated with proteins and is lost upon their denaturation. Interestingly, a decline in antioxidant activity was also detected in the lysates, even though the ABTS assay is generally considered to primarily reflect the presence of low-molecular-weight antioxidants. This observation suggests that a fraction of the antioxidant potential detected in the lysates originates from protein-associated phenolic or other macromolecular components that are removed or inactivated during heating. Together, these data indicate that PEF-assisted enzymatic hydrolysis predominantly releases low-molecular-weight, thermally stable bioactive compounds, in contrast to extracts obtained by mechanical disruption, whose functional properties partly depend on thermolabile macromolecular components.

From an application perspective, the thermal stability of the extracts is advantageous, as it suggests that their functional properties may be preserved during common industrial processes such as pasteurization, drying, or incorporation into thermally processed food matrices. This supports their potential use as functional food ingredients, nutraceutical components, or antioxidant additives.

In this study, we present an alternative approach for the valorization of spent brewer’s yeast based on PEF treatment in continuous-flow mode. The procedure is technically simple and relies on irreversible electropermeabilization of yeast cells suspended in water. The use of water as both the treatment and post-pulse incubation medium at the first stage of the extraction process has several practical implications. First, it simplifies the permeabilization step, thereby reducing overall process complexity. During pulsing, the release of intracellular ions increases the suspension conductivity; however, under the selected conditions (low initial conductivity of the cell suspension and an appropriate combination of electrical parameters), this did not result in excessive temperature rise due to Joule heating, even at this relatively high cell concentration (63 ± 3.7 g DCW/L). Under optimal electrical conditions leading to over 98% irreversible electropermeabilization, the outlet temperature remained within the range of 36–38 °C. Consequently, no temperature adjustment before or after electrical treatment was necessary, a step that is often included in electroporation protocols.

The specific treatment energy required for cell permeabilization (62.9 kJ/L of cell suspension, ≈1 MJ/kg DCW) was relatively low and substantially below the energy demand typically associated with mechanical disintegration methods. Furthermore, PEF in continuous-flow mode allows flexible operation and can be readily adapted to process different volumes of cell suspension. As shown in this study, a four-fold increase in flow rate was achieved by adjusting only the pulse repetition frequency to maintain the same total treatment time.

Following PEF treatment, release of a large fraction of low-molecular-weight, water-soluble intracellular bioactive compounds was achieved by simple incubation of permeabilized cells at room temperature within a relatively short time. Importantly, the process does not require the use of buffers, the addition of salts, or other chemical components. While such conditions are employed by some research groups to enhance extraction efficiency from electropermeabilized cells, they inevitably increase process complexity and cost and may limit the downstream applicability of the obtained fractions, particularly when intended for use as health supplements or food additives. In addition, incubation of the permeabilized cells in water resulted in a low purine nucleotide content, which is a major limitation for the use of yeast extracts in larger quantities for human consumption.

Spent brewer’s yeast obtained after serial repitching is biologically unstable and therefore requires rapid inactivation prior to disposal or reuse because of its high enzymatic activity and susceptibility to microbial contamination. Thermal inactivation is commonly applied; however, it may lead to the loss of heat-sensitive bioactive compounds, including vitamins [69]. In this context, PEF treatment represents an alternative non-thermal inactivation strategy, a well-established effect of electric field exposure [29,32,33].

Even in the absence of subsequent extraction steps, irreversible electropermeabilization inactivates the cells and promotes water release from the biomass, thereby reducing the energy demand for subsequent drying. Brewer’s yeast is widely distributed in the form of inactivated whole cells (e.g., tablets or powders) as a dietary supplement rich in B vitamins, amino acids, and antioxidants. In this context, the resulting aqueous extracts, enriched in low-molecular-weight bioactive compounds, offer the advantage of providing these valuable components without the negative effects associated with the yeast cell wall or the high content of purine nucleotides, and can be used directly or further processed depending on the intended application.

PEF pretreatment markedly enhances the enzymatic hydrolysis of spent brewer’s yeast by the endoprotease Alcalase, yielding extracts with a solid content corresponding to over 51% of the initial cell biomass after 6 h of incubation. The protein and FAN contents of these extracts are comparable to those reported for extracts obtained after 48 h incubation of PEF-treated cells at 37 °C [70]. This high-efficiency arises from the combined effects of PEF-induced loss of plasma membrane barrier function and increased cell wall permeability, together with the relatively small molecular size of Alcalase, which facilitates its access to intracellular and membrane-associated proteins. Because yeast cell wall permeability is largely controlled by the outer mannoprotein layer, these structural components are likely to be at least partially susceptible to Alcalase, which may further increase wall permeability, thereby facilitating enzyme access to intracellular proteins.

In addition, the combined approach presented in this study provides a highly efficient, solvent-free route for the recovery of both soluble and bound phenolic compounds, thereby contributing to the valorization of spent brewer’s yeast as a source of bioactive ingredients. Together with the peptide fraction, the resulting extracts exhibit high antioxidant activity, supporting their potential use in food, nutraceutical, and cosmetic applications. In the present study, enzymatic hydrolysis was performed directly after PEF treatment. However, since a substantial fraction of proteins, phenolic compounds, and nucleic acids remains retained within permeabilized cells during incubation in water, hydrolysis may also be applied after removal of the aqueous fraction containing low-molecular-weight bioactive compounds.

In practice, the release of most soluble components into the aqueous phase occurs within 1–2 h of incubation of electropermeabilized cells in water. Enzymatic hydrolysis may therefore be initiated immediately thereafter, or the recovered cell biomass may be stored for subsequent processing. Such a cascade treatment could enable the selective recovery of different intracellular components and the production of extracts with tailored compositions and functionalities, thereby improving the utilization of the bioactive potential of this residual biomass. In our study, Alcalase was selected to evaluate the applicability of the combined PEF-assisted enzymatic treatment for the efficient production of peptide-enriched extracts with high antioxidant activity. By altering membrane integrity and increasing cell wall permeability, this pretreatment also has the potential to enhance hydrolysis using various exogenous enzymes, depending on the intended application of the obtained extracts.

Overall, PEF treatment appears to be a promising, energy-efficient, and scalable approach for the valorization of spent brewer’s yeast, ranging from simple applications such as cell inactivation and partial dewatering of the yeast biomass to more advanced cascade extraction processes based on the combination of PEF treatment and enzymatic hydrolysis. Further optimization of the procedure may be achieved by refining selected processing steps, including evaluation of the feasibility of reducing washing cycles prior to PEF treatment, particularly with respect to intended downstream applications, increasing the cell mass-to-enzyme ratio, and processing more concentrated cell suspensions. Furthermore, more detailed characterization of the composition and bioactivity of both aqueous extracts and enzymatic hydrolysates could help to demonstrate their functional value and practical potential as dietary supplements and functional ingredients for cosmetic and pharmaceutical applications, thereby supporting the technological relevance of this approach.

## Data Availability

The original contributions presented in this study are included in the article. Further inquiries can be directed to the corresponding author.
